# Selling Sickness or Helping Patients in the Age of Artificial Intelligence

**DOI:** 10.3390/dj14060341

**Published:** 2026-06-03

**Authors:** Melody Moezzi, Bjørn M. Hofmann

**Affiliations:** 1 Lilleaker Tannklinikk, Lilleakerveien 31, 0283 Oslo, Norway; post@lilleaker-tannklinikk.no; 2 Centre for Medical Ethics, University of Oslo, P.O. Box 1130, N-0318 Oslo, Norway; 3 Department for the Health Sciences, Norwegian University of Science and Technology, P.O. Box 1, N-2802 Gjøvik, Norway

**Keywords:** artificial intelligence, dental radiology, commercialization, professional ethics, overdiagnosis, nudging, informed consent, diagnostic inflation

## Abstract

**Background/Objectives**: Artificial intelligence (AI) is increasingly being integrated into dental diagnostics, promising improved detection, efficiency, and patient communication. While these developments offer potential clinical benefits, emerging commercial applications raise important ethical concerns. This study explores how providers of diagnostic AI systems frame their technologies in marketing materials, with particular attention to features designed to influence patient acceptance and increase revenue. **Methods:** An exploratory qualitative thematic analysis was conducted on publicly available promotional content from leading dental AI companies between September and October 2025. Materials were analyzed for recurring rhetorical strategies related to commercialization, persuasion, technological authority, and representations of objectivity. Ethical interpretation was guided by principlism, professional ethics, and virtue-based perspectives. **Results:** The findings show that AI is frequently marketed not only as a diagnostic aid but also as a tool for boosting case acceptance, return on investment, and practice expansion. Visualizations and performance metrics are used rhetorically to position AI as authoritative and objective, encouraging patient compliance while downplaying uncertainty and potential harm. These practices risk undermining patient autonomy, promoting diagnostic inflation and overtreatment, and compromising professional integrity by shifting attention from patient welfare toward commercial outcomes. **Conclusion:** Pervasive marketing of diagnostic AI amplifies existing tensions between professional integrity and commercial incentives in dentistry. Without appropriate safeguards, AI risks reinforcing a transactional model of care in which patients are treated as consumers and diagnostics become instruments of persuasion. To preserve trust and ethical practice, dentists and professional organizations must ensure that AI remains a supportive clinical tool rather than a commercial device, prioritizing transparency, informed consent, and patient-centered care.

## 1. Introduction and Background

Artificial intelligence (AI) is rapidly transforming multiple fields in medicine, and dentistry is no exception. In dental radiography, AI tools promise to enhance clinicians’ ability to detect caries, assess bone levels, segment tooth structures, and highlight subtle findings that may escape the human eye. While these developments offer clear benefits, they also introduce less visible but equally significant challenges. Increased sensitivity and specificity may enable earlier interventions and improved outcomes, but they may also contribute to detection of conditions that are too mild, too uncertain, or clinically irrelevant to warrant treatment, thereby increasing the risk of overdiagnosis and overtreatment [1,2]. In this paper, “diagnostic inflation” refers to the expansion of diagnostic thresholds or categories in ways that increase the likelihood of intervention without clear clinical necessity, as described in the literature on diagnostic expansion and overdiagnosis [1,3].

Clearly, AI can reveal subtle anomalies that might otherwise be overlooked. As human beings, and especially in the reality of a busy clinical practice, dentists cannot diagnose every possible detail of a radiograph with perfect accuracy. Perceptual limitations [intra- and inter-observer variability], time pressure, and fatigue generate omission errors are a genuine risk [4]. Moreover, AI has proven to be able to identify potential pathological patterns that are hardly visible for dentists. Thus, AI can provide a valuable safeguard by ensuring a more systematic overview and by highlighting areas that warrant closer inspection. Yet this very strength introduces a paradox: the ability to “see more” also raises the danger of seeing too much. Findings of uncertain clinical significance may be flagged as suspicious, encouraging unnecessary interventions. This dynamic reflects the technological imperative [5], the assumption that what can be detected ought to be acted upon. Left unexamined, such an imperative risk drives diagnostic inflation, overtreatment, and erosion of professional integrity [4,6]. A related challenge with AI is overreliance and de-skilling, i.e., that clinicians become so reliant and dependent on AI systems that their clinical skills deteriorate or are not developed in the first place: that is, non-skilling [7,8].

While the issue of balancing these potential benefits and harms of AI is gaining increased and warranted attention [5,9], other transformative aspects of AI in dentistry have received less consideration: for example, the use of AI to explain treatment options to patients in ways that may both streamline consent processes and persuade patients into more invasive or extensive treatments, e.g., trough nudging. We now increasingly observe the use of AI as a sales instrument to increase treatments and enhance revenues. As such, this induces a kind of “technological authority” which can be defined as the tendency to attribute objectivity and credibility to technological outputs, such as AI-generated information, which may reduce critical scrutiny by both clinicians and patients [7].

This expansion from diagnostic empowerment to sales instrument raises specific profound ethical concerns. At stake is the trust towards and the professional integrity of the dentist: are we health professionals guided by patients’ welfare and professional norms or service providers with profit goals? This tension was also the focus of our article “Healthcare Professional or Salesperson? An Examination of the Role of the Dental Profession in the Light of Contract Theory and Normative Ethics” [10] and has likewise been discussed by other scholars who have examined how commercialization and conflicting obligations challenge professional integrity in dentistry [10,11,12,13].

AI may intensify this basic conflict of interest in dentistry and raises fundamental ethical questions concerning professional responsibility, transparency, and respect for patient autonomy. More specifically, it highlights risks associated with the commercial framing of diagnostics, patient influence and persuasion, erosion of informed consent, diagnostic inflation, overtreatment, and compromised professional integrity.

In this work, we examine how the introduction of AI enhances commercialization in the private dental health service and the intensified commodification of dental treatments risk transforming patients into consumers, dental treatments into commodities, and dentists into salespersons. Such a development promotes a transactional rather than a healing relationship and alters the social contract that defines dental professions. As the field adapts to an open market with accentuated revenue demands, there is a growing risk that dentistry will drift away from its traditional role as a health profession bound by a social contract in which the patient’s best interests are paramount.

By doing so, we reveal the paradoxical nature of AI. On the one hand, AI can strengthen diagnostic accuracy, support clinical decision-making, and enhance patient understanding [9,14]. On the other, the way AI is marketed, emphasizing treatment acceptance, revenue growth, and reduced patient skepticism, creates ethical challenges that echo, and even intensify, the previously described concerns of commercialization [10,11,12,13]. The term “selling sickness” stems from the debate on disease mongering [15], and is used here to describe practices that expand the conception of disease in ways that increase demand for treatment or care, often by lowering diagnostic thresholds or emphasizing risk.

Accordingly, the objective of this article is to provide an ethical analysis of using AI to persuade or nudge patients to have more extensive examinations and treatments than professionally necessary. The research questions are:

1. How do providers of diagnostic AI systems use the systems’ patient persuasion features to increase revenues in the promotion of their technology?
2. What potential normative ethical concerns are raised by this practice?
3. How can the strategies identified in this study be ethically evaluated using established frameworks of biomedical and professional ethics?

## 2. Methods

This study is an exploratory qualitative analysis of publicly available marketing material produced by commercial providers of AI-based diagnostic systems in dentistry. The aim was not to provide a comprehensive or systematic mapping of the field, but to identify and analyze recurring patterns in how such technologies are framed and presented, particularly with regard to persuasion, commercialization, and representations of objectivity.

Marketing material was collected from company websites, official social media channels (including Instagram, LinkedIn, and YouTube), and publicly accessible press releases between September and October 2025. The material included promotional texts, images, videos, advertisements, and marketing statements directed at dental professionals. A purposive sampling strategy was applied. Companies were selected based on their international visibility, presence in the dental AI market, and availability of English-language promotional content. The final sample included Pearl AI, Overjet, VideaHealth, and Diagnocat, which are widely referenced actors within the current dental AI landscape.

The dataset consisted of a diverse selection of marketing items across platforms, including website pages, promotional videos, and social media posts. Given the large volume of available material (e.g., several hundred posts on individual company accounts), the selection was guided by relevance rather than exhaustiveness. Materials were included if they contained explicit or implicit references to clinical decision-making, patient communication, case acceptance, revenue, return on investment, efficiency, or practice growth. Materials focusing solely on technical specifications without communicative or rhetorical elements were excluded. The purpose of this approach was to capture dominant and recurring patterns in marketing narratives, rather than to quantify their frequency or distribution.

The collected materials were analyzed using qualitative thematic analysis as described by Virginia Braun and Victoria Clarke [16,17,18]. A reflexive thematic approach was applied, which emphasizes the interpretative role of the researchers in identifying patterns of meaning within the data. In line with Braun and Clarke’s reflexive thematic analysis, themes were understood as interpretative constructions developed through engagement with the data rather than as objective features of the material. The analysis was primarily inductive, allowing themes to emerge from the material, while also being informed by the study’s ethical focus. This combination reflects an abductive analytical strategy, in which empirical observations and theoretical perspectives are iteratively related.

The analytical process followed the phases outlined by Braun and Clarke, including familiarization with the data, initial coding of salient features, generation of candidate themes, review and refinement of themes, and definition and naming of themes. Both explicit content (e.g., references to case acceptance, revenue, efficiency) and implicit rhetorical elements (e.g., appeals to trust, authority, and objectivity) were coded. Both textual and visual elements were included in the analysis. Particular attention was given to the rhetorical function of visualizations, such as radiographic overlays and color coding, and how these were used in relation to claims about objectivity, trust, and clinical decision-making.

The coding and analysis were conducted by the authors and involved continuous discussion and reflexive engagement with the material. In accordance with a reflexive thematic approach, the aim was interpretative depth and conceptual insight, rather than inter-rater reliability or coding consensus [17,18].

Ethical interpretation was conducted as a second analytical step. Identified themes were examined using established frameworks in biomedical ethics, including the principles of autonomy, beneficence, non-maleficence, and justice [19], as well as perspectives from professional ethics, social contract theory, and professional integrity [11,12,13]. This two-step approach allowed for a clear distinction between empirical observations (how AI is framed in marketing materials) and normative analysis (how these framings may be ethically evaluated in relation to patient autonomy, informed consent, professional responsibility, and trust).

As the study is exploratory and interpretative in nature, saturation was not its aim. Moreover, as the analysis did not aim for statistical generalization, no formal assessment of inter-coder reliability was performed. As mentioned, the purpose of the study was not to provide a comprehensive empirical mapping, but to generate conceptual and ethical insight into emerging patterns in the commercial framing of AI in dentistry. The consistency of themes across multiple providers and platforms supports the relevance of the findings despite these limitations.

## 3. Results

Materials from Pearl AI, Overjet, VideaHealth, and Diagnocat were included, as these are leading international actors with publicly available promotional materials.

### 3.1. Marketing and Rhetoric

As one of the leading commercial providers in this field, Pearl AI’s promotional material illustrates how artificial intelligence is framed not primarily as a diagnostic support system, but as a commercial and rhetorical tool. Across advertisements, social media, and its website, Pearl AI repeatedly emphasizes treatment acceptance and revenue growth as central selling points. Banners highlight statistics such as “Boost Case Acceptance with AI” with claimed increases of +37% for scaling and root planing, +34% for crowns, and +19% for fillings. Other marketing promises include “Grow your practice,” “Guaranteed ROI,” and claims that Pearl’s AI is “the most powerful chair-side AI platform.” More provocatively, marketing collateral asserts that AI visualizations make it “harder for patients to question” the treatment plan [20,20]. In such framing, AI easily goes beyond being a diagnostic assistant and becomes a tool of persuasion.

Overjet, another leading commercial actor, similarly frames its AI system in terms of practice growth, financial return, and regulatory legitimacy. Promotional materials highlight metrics such as “25% higher care acceptance,” “18× average ROI,” and significant efficiency gains in clinical workflows, alongside repeated references to FDA clearance as a marker of credibility [21,21,21]. Dedicated marketing content focuses explicitly on “The ROI of Overjet,” targeting dental service organizations (DSOs) and emphasizing that the system will “pay for itself” by increasing treatment acceptance and production. In this context, ROI is not presented as a secondary benefit but as a central rationale for adoption, positioning AI as a financial investment rather than primarily a patient-safety or diagnostic-quality tool.

VideaHealth emphasizes rapid integration and case acceptance, often presenting its technology to “unlock hidden revenue” in existing radiographs [22,22]. VideaHealth provides a guide of four “refreshingly simple steps to boost patient case acceptance” including “prep the patient and promote AI” and “don’t just share the diagnosis—show it”, with the claim that “AI can help increase trust, streamline diagnosis, and boost case acceptance”. They also platform copy about “maximize production,” “scale smarter,” and “add daily production.” The system provides a “second set of eyes” to catch issues earlier, and claims of 20%+ acceptance lift from customers [22].

Diagnocat, which has a stronger foothold in the European market, similarly combines clinical and commercial messaging. While the system is presented as an advanced, comprehensive AI tool capable of detecting multiple pathologies from radiographic data, promotional materials also emphasize efficiency gains, workflow optimization, and practice differentiation [23,23]. Outreach materials highlight how automation and faster reporting can increase productivity and reduce costs, thereby indirectly linking diagnostic AI to financial performance and competitive advantage.

Across these examples, AI is framed not only as a tool for clinical accuracy but also as a driver of compliance, profitability, and business success. Such rhetorical strategies appeared consistently across the reviewed material and across multiple providers.

The overview and examples of the findings with respect to commercial and/or ROI framing versus patient-centered and/or clinical framing and associated ethical risks are shown in Table 1.

### 3.2. Normative Ethical Analysis

The findings also point to a set of recurring ethical challenges associated with the commercial framing of AI in dentistry. These include concerns related to autonomy, such as persuasive framing that may reduce critical reflections and increase the risk of overtreatment. They further reflect a potential shift from beneficence toward profit, including diagnostic inflation and commercial pressure in clinical decision-making. In addition, the material suggests the use of communication strategies that blur the line between informing and persuading, potentially increasing treatment uptake without clear necessity. Finally, elements of commercial bias are evident, including subtle pressure toward intervention and the prioritization of efficiency and competitiveness over patient-centered deliberation. In particular, the material challenges four basic principles of biomedical ethics, i.e., autonomy, beneficence, non-maleficence, and justice.

### 3.3. Autonomy

As the examples illustrate, the principle of autonomy may be infringed, as patient decision-making may be shaped more by persuasion than by balanced information. The consequence is that the patient does not give real or valid informed consent. The line between persuasion and manipulation is fine, and AI systems may bias the understanding of the patient, which is a key requirement for consent and autonomy. For example, Pearl AI’s ads emphasize that AI visualizations make it “harder for patients to question” treatment plans and can “close the trust gap.” Rather than supporting patient understanding in a neutral manner, AI is presented as a tool to overcome skepticism and encourage acceptance. This shifts the communicative purpose from informing to persuading, raising concerns about patient autonomy and informed consent [8].

Correspondingly, the marketing language invests technology with an aura of authority and truth, lending itself to paternalism. Claims such as “AI you can stand behind” or “dentistry’s most powerful AI platform” portray AI as infallible and objective. Similarly, color overlays on radiographs presented in ads give the impression of clinical certainty, when in fact they are algorithmic interpretations. This rhetorical use of technology strengthens the dentist’s authority while simultaneously reducing the patient’s space to question or seek alternatives [8,24,25].

Relatedly, the aura of technological objectivity of the AI systems may obscure the inherent limitations and uncertainties of AI systems. When AI outputs are presented as “objective evidence,” uncertainty may be downplayed, reducing openness about false positives, false negatives, and the interpretive character of algorithmic overlays. Hence, AI systems may reduce transparency and patients’ understanding, and thereby undermine real informed consent and patient autonomy.

In general, while nudging is classified as libertarian paternalism [26], it still is a kind of paternalism that challenges autonomy [27].

### 3.4. Beneficence

Unnecessary examinations or treatments reduce the benefit to the patient and risk that economic incentives and persuasive technology will overshadow the dentist’s duty to act in the patient’s best interests. This can be seen in Pearl AI’s promotional strategy to promise financial gain. Marketing messages such as “Grow your practice” and “Guaranteed ROI” frame the software not primarily as a clinical tool, but as a business investment. This leaves the impression that patients are implicitly cast as revenue sources, and successful dentistry is equated with increased production and profitability. This commercial framing shifts attention away from the dentist’s role as a health professional with a trusted duty to act in the patient’s best interests, and toward the role of a salesperson whose success is measured in economic return [11,12]. Similarly, Overjet’s focus on treatment acceptance in ROI shifts the emphasis from beneficence for the patient to the economic benefit of the professional.

In addition, increased case acceptance rates can result in overdiagnosis, diagnostic inflation, and overtreatment where “seeing too much” risks encouraging unnecessary interventions.

### 3.5. Non-Maleficence

Unnecessary examinations or treatments increase the chance of harm. In this context, potential harms include overdiagnosis, iatrogenic damage from avoidable interventions, increased patient anxiety, and downstream overtreatment triggered by uncertain or inflated findings. None of the providers included any information on this. They did not provide relevant information of any of the other challenges with AI systems, such as bias, hallucination, model drift, de-skilling, and lack of explainability, either.

### 3.6. Justice

Directing attention and resources from patient needs and toward revenue-generating treatments has opportunity costs and may skew access to care. In addition, persuasive AI-based communication may disproportionately influence vulnerable patients with limited health literacy, thereby exacerbating inequities in decision-making and outcomes. In sum, the use of AI systems that is identified in this study may substantially contribute to undermining just access to healthcare services.

### 3.7. Professional Integrity

In addition to the infringement of the four basic ethical principles, the AI promotions revealed in this study undermine professional integrity. This challenges the profession’s social contract and undermines long-term trust in the profession. Hence, when AI is marketed as a sales tool, the dentist’s identity as a health professional is compromised [11,12].

### 3.8. Technological Persuasion

A further ethical challenge arises from the rhetorical status of technology. AI-based visualizations often carry an aura of objectivity and authority, giving the impression that what is displayed is equivalent to truth [5]. This technological status can bias both patients and clinicians: patients may feel compelled to accept treatment because “the computer shows it,” while dentists may place undue trust in AI outputs at the expense of their own clinical judgment [11,12]. Technological authority potentially discourages critical judgment by both patients and clinicians.

### 3.9. Selling Sickness

The findings (Table 1) tend to fall under what has been called “disease mongering” and “selling sickness”. The term “selling sickness” has been widely used to describe how pharmaceutical companies, medical device manufacturers, and parts of the healthcare industry promote disease awareness campaigns or diagnostic criteria in ways that expand markets for their products [15,28,29]. Rather than focusing solely on improving patient health, such strategies are designed to increase demand for treatment, sometimes by redefining the boundaries of illness, lowering thresholds for diagnosis, or emphasizing worst-case scenarios. This phenomenon has been critically analyzed in medicine as a driver of overdiagnosis, overtreatment, and medicalization of normal human experiences. By drawing on this concept, we suggest that similar dynamics may now be visible in dentistry, particularly in the way AI tools are marketed.

## 4. Discussion

Our findings indicate that providers of diagnostic AI systems use the systems’ patient persuasion features to increase uptake and revenues in the promotion of their technology in a range of rhetorical ways. Moreover, we have pointed out that this raises a series of normative ethical issues, infringing basic ethical principles, such as autonomy, beneficence, non-maleficence, and justice, as well as undermining professional integrity [10,11,12,13].

In this study, we have applied a principlist approach to bioethics. Additionally, normative ethical theories can provide complementary lenses in examining AI marketing in dentistry through deontological duties, consequentialist outcomes, virtue ethics [30], and the perspective of professional ethics and social contract theory [10,12]. For example, deontological analysis highlights the breach of duties toward truthfulness and autonomy, while consequentialist reasoning underscores the likelihood of negative outcomes when commercial rather than clinical benefits are emphasized. Virtue ethics exposes how such practices risk reshaping the moral character of the profession, cultivating opportunism rather than integrity [30]. Finally, professional ethics and social contract theory demonstrate how marketing framed around revenue and persuasion jeopardizes the very trust upon which dentistry’s legitimacy rests [10,11,12,13]. Altogether, we can better understand not only the risks at stake but also the kind of professional identity and moral integrity that should guide the dental profession in the face of technological and commercial pressures.

Taken together, these perspectives show that the ethical challenges identified are not confined to isolated marketing practices or individual actors but reflect broader structural tensions between professional obligations and commercial incentives in contemporary dentistry [10,11,12,13]. By framing AI primarily as a means to increase treatment uptake, Pearl AIs marketing risks amplifying dynamics of “selling sickness” already described in the medical literature [15,28,29]. Instead of empowering patients with clearer information, the rhetoric suggests using technology to reduce skepticism and close the “trust gap,” thereby shifting communication from dialog toward persuasion.

Similar concerns have been raised in other areas of medicine, such as radiology and dermatology, where AI-supported diagnostics and decision tools may influence clinical decision-making and patient communication in ways that increase intervention rates [31,32,33,34].

The way AI is presented to dentists sets the stage for how it will be used in practice. Marketing that emphasizes “guaranteed ROI,” “boosted case acceptance,” and making it “harder for patients to question” treatment plans redefine the purpose of diagnostics [10,11,12,14,15,16,17]. Rather than safeguarding accuracy and patient safety, radiographs become positioned as persuasive tools to secure patient compliance and generate revenue. This reframing undermines professional duties and contributes to a culture in which commercial success is prioritized over patient welfare [10,11,12,13,14,15].

Beyond the marketing rhetoric, the integration of AI into daily clinical practice also raises profound ethical challenges. On the one hand, AI can improve detection, reduce omission errors in busy settings, and support more systematic evaluations [3]. On the other hand, these same strengths create new vulnerabilities. By flagging subtle or uncertain anomalies, AI may contribute to diagnostic inflation and overtreatment [1].

Further concern lies in the biases embedded in AI systems themselves. As Hofmann has argued, bias in AI is not an exception but an inevitable feature of how such systems are designed and trained [5]. Algorithms are shaped by the data they rely on and the assumptions of their developers. In dental radiography, this may mean that AI over-detects certain patterns, under-represents others, or reflects the commercial priorities of its producers [32]. Such biases can distort diagnostic judgment and reinforce the tendency to “see too much,” thereby compounding risks of overtreatment and unnecessary intervention.

What emerges, therefore, is a convergence of risks: marketing rhetoric that encourages a commercial mindset and algorithmic dynamics that reinforce diagnostic expansion. If left unaddressed, these forces may normalize a view of dentistry in which patients are primarily consumers, diagnostics function as tools of persuasion, and clinical decisions are subtly shaped by economic rather than ethical considerations [5,10,11,12,13,32,34].

It is important to notice that we do not deny the potential benefits of AI for diagnoses and treatments in dentistry. We do not reject the idea that AI systems can be used for promoting appropriate interventions, e.g., by providing convincing information to reluctant patients. The point in this article is rather the ethically challenging use of such systems.

Importantly, these risks are compounded by the authoritative status often attributed to AI-generated visualizations. When algorithmic overlays are perceived as objective or definitive, both patients and clinicians may be less inclined to question their validity or relevance in individual cases [5].

To prevent such a development, the profession must take responsibility for how AI is adopted and communicated in practice. Dentists should insist on transparency regarding the limitations and uncertainties of AI outputs, presenting them as supportive tools rather than as unquestionable truths [5]. Patients must be given balanced information that emphasizes alternative options, the possibility of watchful waiting, and the provisional nature of many radiographic findings [1,2]. Dentists should also remain vigilant about their own use of language, avoiding sales-oriented framings that reduce the space for genuine dialog [10,11,12,13,14,15]. Professional organizations and educators can play an important role by providing training in the ethical use of AI, including awareness of biases and the risks of over-reliance [5,8,24,25]. Finally, clinics should consider internal safeguards, such as peer discussions, second opinions, or regular audits of treatment patterns, to ensure that AI is serving patient welfare rather than production goals. Please see the suggested checklist in Box 1 for details.

Box 1 **Checklist for the ethical use of AI in dental practice.**

• **Frame AI correctly**: Present it as an aid to support your diagnosis, not as a final authority.
• Explain limitations: Tell patients that AI highlights possible findings but can both miss and over-detect.
• **Balance the options**: Always include preventive or observational alternatives, not just operative treatments.
• **Avoid persuasive language**: Focus on health outcomes, not “acceptance rates” or financial return.
• **Check your own judgment**: Critically review AI outputs before discussing them with the patient.
• **Safeguard autonomy**: Invite questions and emphasize that the patient has the right to decline or seek a second opinion.
• **Be alert to bias**: Remember that AI is shaped by training data and may reflect systematic distortions.
• **Document carefully**: Note in the record how AI was used and how treatment decisions were reached.
• **Beneficence**: Prioritize patient benefit over professional financial gain.

Taken together, these steps can help ensure that AI strengthens rather than undermines the ethical foundations of dentistry. By resisting the pressure to use new technologies as sales instruments, dentists can uphold their professional integrity and maintain the trust that is essential for the healing relationship at the core of their vocation.

As with all research, this study also has its weaknesses. First and foremost, this is not a systematic review of the marketing. The search was exploratory and limited to publicly accessible material in English. The focus was on identifying recurring rhetorical and ethical patterns, rather than measuring frequency or representativeness. Nevertheless, the findings reveal consistent trends across several major commercial AI systems, suggesting that the persuasive marketing of AI in dentistry is a broader phenomenon, rather than an isolated case. Moreover, we have applied a purposive sampling strategy limited to publicly available material in English, which may introduce selection bias. However, readers may peruse our findings and compare them to other findings to assess the transferability of our results.

## 5. Conclusions

Artificial intelligence has the potential to improve diagnostic accuracy and patient care in dentistry, but its current marketing as a sales instrument to increase return of investment threatens to infringe basic ethical principles and undermine professional integrity and public trust. By framing AI around case acceptance and revenue, commercial actors risk amplifying dynamics of “selling sickness” that have been long criticized in medicine.

When AI-based visualizations are presented as authoritative or objective, they may further reinforce persuasive uses of diagnostics at the expense of critical judgment and informed consent. As such, AI systems enhance the dentist’s conflict of interest between providing the best health outcomes for the patient and increasing revenues.

Dentists, professional organizations, and educators must therefore adopt safeguards to ensure AI remains a supportive clinical tool rather than a persuasive device. Only by prioritizing patient welfare over profit can the profession integrate AI ethically while maintaining its integrity, trust, and social legitimacy.

## Figures and Tables

**Table 1:** Overview and examples of the findings with respect to commercial and/or ROI framing versus patient-centered and/or clinical framing.

Vendor	Commercial/ROI Framing	Patient-Centered/Clinical Framing
Pearl AI	“Boost case acceptance” messaging and tips aimed at increasing acceptance rates and practice outcomes. Press materials highlight partnerships to “improve case acceptance” and patient communication using visual mapping/overlays.	Claims that AI provides “objective, chairside evidence,” strengthens communication, improves transparency/trust, and thus improves patient care and understanding. Blog posts argue AI helps clinicians “step back and assess patient care,” improving engagement and clarity of plans.
Overjet	Prominent performance metrics: “25% higher care acceptance,” “18× average ROI,” efficiency gains [hours saved/week]. Dedicated materials on “The ROI of Overjet,” DSO analytics, production value focus; blogs linking AI to production/ROI levers.	Brand narrative: “Put patients first,” “improve oral health outcomes,” align dentists and payers to benefit patients; improved engagement, diagnostic accuracy, outcomes. “Patient experience” posts emphasize clearer communication and smoother journeys.
VideaHealth	Guides explicitly about “boost patient case acceptance”; platform copy about “maximize production,” “scale smarter,” and “add daily production.”	“Build trust and increase case acceptance” via clear visuals; “second set of eyes” to catch issues earlier; claims of 20%+ acceptance lift from customers.
Diagnocat	Event copy references practice differentiation, streamlining processes, reducing costs, “benefiting… practice.”	Site/blog emphasize supporting decision-making, enhancing patient understanding, accurate diagnoses, clinician retains final decision.

## Data Availability

The original contributions presented in this study are included in the article. Further inquiries can be directed to the first author.
